# Optimizing Visualization of Pollen Tubes in Wheat Pistils

**DOI:** 10.3390/plants13243600

**Published:** 2024-12-23

**Authors:** Kohei Mishina, Minami Morita, Sora Matsumoto, Shun Sakuma

**Affiliations:** Faculty of Agriculture, Tottori University, Tottori 680-8553, Japan; mishina@tottori-u.ac.jp (K.M.);

**Keywords:** pistil, pollen tube, wheat, wide hybridization

## Abstract

Successful pollination and fertilization are crucial for grain setting in cereals. Wheat is an allohexaploid autogamous species. Due to its evolutionary history, the genetic diversity of current bread wheat (*Triticum aestivum*) cultivars is limited. Introducing favorable alleles from related wild and cultivated wheat species is a promising breeding strategy for resolving this issue. However, wide hybridization between bread wheat and its relatives is hampered by the presence of suppressor genes and difficulties in crossing. Optimized methods for observing pollen tubes are essential for understanding the mechanism of crossability between wheat and its relatives. Here, we improved the crossing procedure between bread wheat and rye (*Secale cereale*) and established an optimized protocol for visualizing pollen tube behavior. Crossing via detached spike culture significantly enhanced crossing efficiency and phenotypic stability. A combination of canonical aniline blue staining and optimized clearing and sectioning allowed us to visualize pollen tube behavior. The proportion of rye pollen tubes reaching the micropyle was lower than that for pollen tubes germinated on the stigmatic hair, explaining why the hybrid seed-setting rate was approximately 75% instead of 100%. This method sheds light on wide hybridization through deeper visualization of the insides of pistils.

## 1. Introduction

Bread wheat (*Triticum aestivum*) is the most important crop worldwide, providing approximately 20% of calories and protein in the human diet [1]. Global wheat production in 2024–2025 is estimated at 792.9 million tons, accounting for 27.8% of worldwide cereal production (https://www.fao.org/worldfoodsituation/csdb/en (accessed on 18 November 2024)). Meeting the demand for bread wheat in an environmentally and socially sustainable manner has become an unprecedented challenge under climate change, which affects yield and stability [2]. Therefore, breeding wheat varieties with improved resilience and resistance to environmental stress is essential.

Bread wheat is an allohexaploid species (AABBDD genome; 2*n* = 6*x* = 42) that arose from two ancient interspecific hybridization events. The first event occurred 0.36–0.5 million years ago between the diploid species *Triticum urartu* (AA genome) and *Aegilops speltoides* or an extinct, closely related *Sitopsis* species (SS or BB genome). This hybridization gave rise to the tetraploid species *Triticum turgidum* (AABB genome). The second event occurred ~7000 years ago and involved domesticated tetraploid wheat and the D genome donor *Aegilops tauschii,* leading to hexaploid bread wheat. In the context of wheat evolution, wide hybridization with wild relatives is key for introducing favorable alleles that are lacking in wheat cultivars [3,4,5]. For example, introgression of *Triticum dicoccoides* genes provides environmental adaptation and agronomic trait diversity [6], *Ae*. *tauschii* genes provide heat tolerance to wheat [7], *Thinopyrum elongatum* genes provide disease resistance against Fusarium species [8], *Secale cereale* genes provide tolerance of drought [9], and *Leymus racemosus* genes provide tolerance of low nitrogen fertilizer input [10]. The highly crossable genotype Chinese Spring can be hybridized to *Aegilops* [4,5], rye (*Secale cereale*) [11,12], barley (*Hordeum vulgare*) [13], and other plants [14].

Floret fertility, determined by floral organ development and the success of pollination and fertilization [15], is a key determinant of the number of grains per inflorescence [16]. Bread wheat has a bisexual floret consisting of three anthers, one pistil, and two lodicules [17]. The rate of self-pollination is estimated to be 96.5–100%, and thus this crop is autogamous in nature [18]. A small amount of outcrossing occurs due to asynchronous flowering; the mismatch between pollen dehiscence and stigma collapse within a floret is approximately 30% at anthesis [19]. Pollen viability is significantly reduced at temperatures >30 °C, thus also promoting outcrossing [20].

Pollen staining can be used to examine starch and chromatin formation [21]. Live/dead pollen can be discriminated by fluorescein diacetate (FDA) or propidium iodide (PI) staining [22]. A raffinose-based in vitro pollen germination medium has been used to observe pollen germination and pollen tube elongation [23]. Despite these advancements in pollen viability testing techniques, uncertainty remains regarding pistil viability and compatibility between pollen grains and pistils, and there is a need to develop an optimized technique for observing pollen tubes in wheat pistils. In this study, we successfully investigated floret fertility following the fertilization of wheat stigmata with rye pollen by developing an optimized technique for observing pollen tube growth through the stigma, style, and ovary. Our findings shed light on the mechanism underlying reduced seed set following wide crossing, as well as confirming the utility of our new technique for investigating fertilization in wheat.

## 2. Results

### 2.1. Improved Method for Wide Crossing

**Figure 1:**
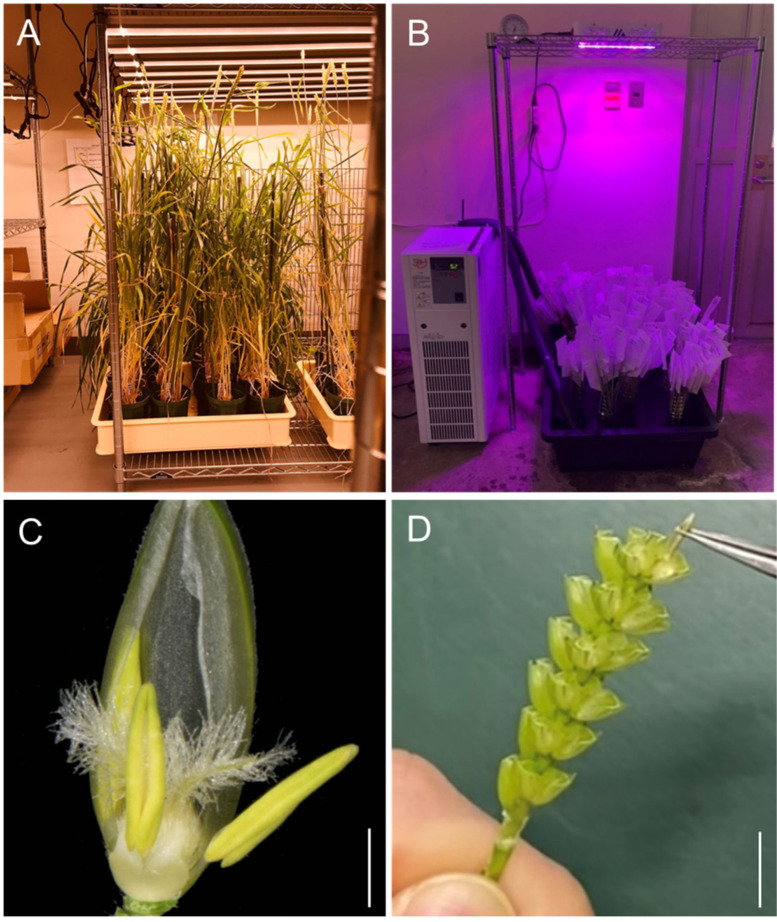
Improved crossing procedure. (**A**) Wheat and rye plants growing in a growth room. (**B**) Incubator for detached spikes with circulating cold water. (**C**) Floret structure of bread wheat. (**D**) Hand pollination of a wheat pistil using rye pollen. Scale bars: 1 mm in (**C**), 1 cm in (**D**).

To ensure successful crossing between common wheat and rye, we used the detached spike culture method from plants grown under controlled conditions (Figure 1A,B). We dissected spikes from common wheat with at least two nodes remaining from the peduncle at an estimated 2–3 days before flowering. For emasculation, we manually removed three yellowish-green anthers from slightly open stigmata (Figure 1C). At 2 or 3 days after emasculation, we hand-pollinated the stigmata with fresh rye pollen (Figure 1D). We were able to perform emasculation and pollination of 20 florets within a spike in only 5 min, allowing us to pollinate approximately 100 spikes per day.

### 2.2. Optimizing Floret Position for Crossing

**Figure 2:**
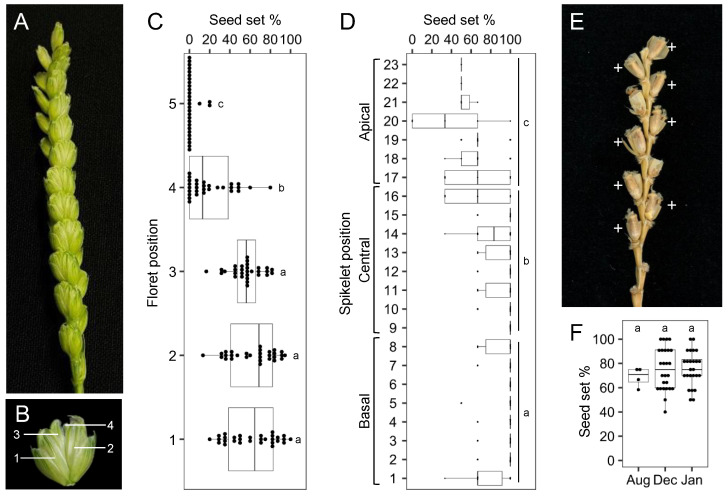
Fertility of wheat × rye hybrid seeds at different floret positions. (**A**) Example of a wheat spike consisting of 24 spikelets. (**B**) Example of a wheat spikelet consisting of five florets. The 5th floret was hidden between 3rd and 4th florets. (**C**) Effect of floret position on seed set based on 30 spikes. (**D**) Effect of spikelet position at the 1st, 2nd, and 3rd florets on seed set based on 30 spikes. (**E**) Example of hybrid seed set using spikelets 3–12, containing 1st and 2nd florets (**F**) Crossability in three independent experiments (growing during summer and winter; *n* = 4 spikes in August, *n* = 26 in December, and *n* = 25 in January). Different letters in (**C**,**D**,**F**) indicate significant differences using Tukey’s multiple comparison test. Box edges represent the 25% and 75% quantiles, with the median values indicated by center horizontal lines. Whiskers indicate 1.5 times the interquartile range.

We compared hybrid seeds generated by pollinating wheat florets at different positions with rye pollen to identify the effective floret position for crossing. We used Chinese Spring wheat, which produces ~24 spikelets per spike (Figure 2A) and 4.45 ± 0.83 (range 3–5) florets within a spikelet (Figure 2B). The 1st–3rd florets showed the highest hybrid seed-setting rates (Figure 2C), whereas the 4th and 5th florets showed lower hybrid seed-setting rates. These results suggest that the 1st and 2nd florets are optimal for crossing and exploring crossability. Higher hybrid seed-setting rates were obtained from the basal and central parts of the spike (spikelets 1–16), whereas the apical part of the spike (spikelets 17–23) had a lower hybrid seed-setting rate (Figure 2D). Floret fertility of self-pollinated wheat in the basal and central spikelets was also high (Figure S1). Therefore, we used the bottom ten spikelets and the 1st and 2nd florets for further experiments. In three independent experiments (seasons), we obtained consistent hybrid seed-setting rates: 68.6 ± 8.0% in August, 75.5 ± 17.2% in December, and 75.9 ± 14.0% in January (Figure 2E,F). These results indicate that the improved methods work effectively in any season, with comparable results.

### 2.3. Optimizing Pollen Tube Visualization

In principle, pollen tubes stained with aniline blue and propidium iodide (PI) should be visible under a fluorescence microscope. We used PI to label pollen grains and pollen tubes [24] and tested modified tissue-clearing and sectioning methods to improve pollen tube visualization.

**Figure 3:**
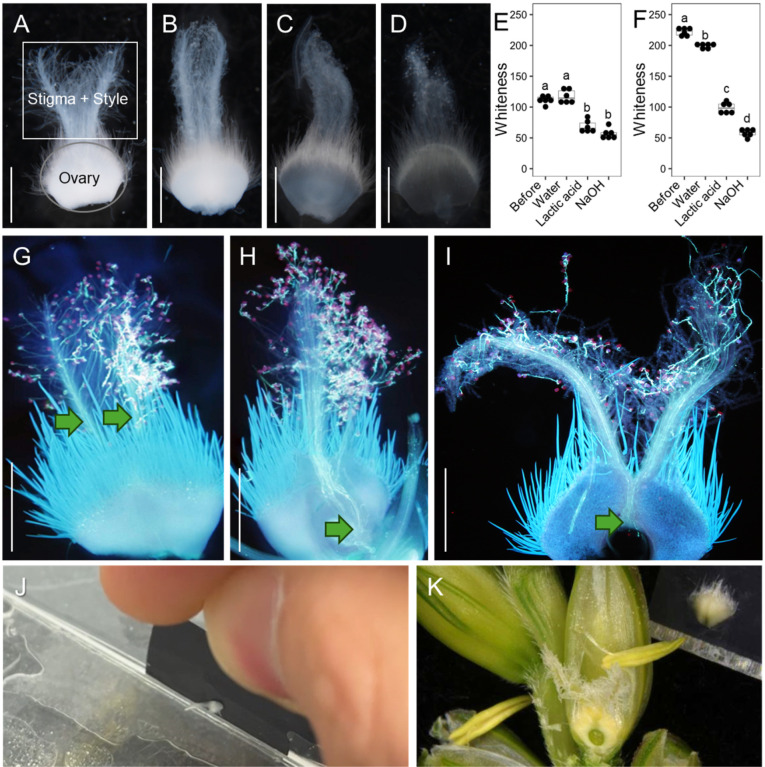
Technical advances in pollen tube observation. (**A**–**F**) Effects of clearing treatments. (**A**) Pistil before clearing treatment. The rectangle and circle indicate the positions where whiteness was measured. (**B**–**D**) Pistils after heating in water (**B**), 70% lactic acid (**C**), and 1 N sodium hydroxide (**D**). (**E**,**F**) Measuring the whiteness of the stigma/style region (**E**) and ovary segments (**F**). The groups consisted of *n* = 6 pistils. Different letters indicate significant differences using Tukey’s multiple comparison test. (**G**–**I**) Comparison of cutting methods using 70% lactic acid clearing, whole pistils (**G**), vertical sectioning (**H**), and horizontal sectioning (**I**). (**J**) The newly designed tool used to generate vertical hand sections of pistils. (**K**) Horizontal sectioning of a pistil. Arrows in (**G**–**I**) indicate pollen tube signals. Scale bars (**A**–**D**,**G**–**I**), 1 mm.

We compared three clearing treatments: 70% lactic acid, 1 M sodium hydroxide, and water (Figure 3A–D). Before treatment, the whiteness levels of the stigma and style were 111.7 ± 6.4, and that of the ovary was 222.1 ± 5.8. Heating treatment was adequate to make the tissue transparent (Figure 3E,F). Heat incubation for 2 min was appropriate; the tissues became too soft when heated for more than 10 min. Both 70% lactic acid and 1 M sodium hydroxide treatment allowed us to distinguish the background autofluorescence of the ovary (Figure 3G–I). After heating in 70% lactic acid, the whiteness level of the stigma and style was 68.6 ± 9.7, and that of the ovary was 98 ± 8.6. After 1 M sodium hydroxide treatment, the whiteness level of the stigma and style was 56.8 ± 8.5, and that of the ovary was 57.8 ± 6.2. However, since 1 M sodium hydroxide treatment removed the red signals from pollen grains, we chose 70% lactic acid as the optimal clearing treatment.

The fluorescence signals from rye pollen tubes were detected in the stigma and style, but not in the ovary, without tissue dissection (Figure 3G) due to the thickness of the ovary (188 ± 18.8 μm) and autofluorescence from ovary hairs. To address this issue, we generated vertical and horizontal sections of the tissue. The tool used for vertical sectioning was developed in-house (Figure 3J). A thin groove in the knife allows it to rotate exactly 90 degrees. Using vertical sections, we successfully detected pollen tube signals in the ovary (Figure 3H). After creating horizontal sections, we removed the ovary hairs and exposed the ovule (Figure 3K). In horizontal sections, we observed pollen tube signals in the upper part of the ovary (Figure 3I).

### 2.4. Investigating the Reduction in Hybrid Seed Production

**Figure 4:**
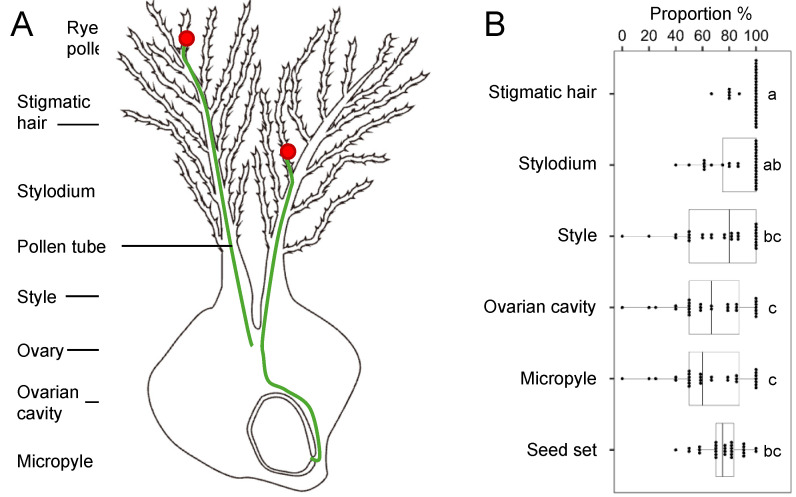
Frequencies of penetrated pollen tubes in the pistil and seed sets. (**A**) Schematic representation of pollen tubes in a wheat × rye cross. Pistil organ terms follow [25]. (**B**) Proportion of pollen tube presence in different regions of the pistil. Results were obtained from 29 spikes as technical repeats. Different letters indicate significant differences, as determined using Tukey’s multiple comparison test.

To determine why the hybrid seed set in Chinese Spring × rye was ~75%, not 100%, we counted the frequency of penetrated pollen tubes (Figure 4A). The frequencies of pollen tubes that germinated on the stigmatic hair, reached the stylodium, and reached the style were 95.7 ± 9.1%, 86.5 ± 18.8%, and 72.8 ± 26.9%, respectively (Figure 4B). The frequencies of pollen tubes that reached the ovarian cavity (66.5 ± 27.4%) and micropyle (65.9 ± 27.3%) were significantly lower. These results suggest that the penetration of rye pollen tubes is suppressed between the stylodium and ovarian cavity of Chinese Spring.

## 3. Discussion

In this study, we developed an improved crossing procedure for wide crossing in wheat, which allowed us to perform more than 100 crosses in a single day. Using this method, the stability of hybrid seed set improved regardless of outdoor conditions. When performing wide crossing, the difference in flowering time between the two parental species can be problematic. For example, in triticale hybrid production, the flowering time of bread wheat ranges from the middle of March to late May, and that of rye ranges from early May to late May in Tottori, Japan. The use of growth rooms makes it relatively easy to circumvent this limitation. The use of detached spike culture also improves working conditions, as emasculation and pollination can be performed while sitting at a lab bench, regardless of the weather. In the field, the pollination procedure is usually disturbed by windy and cloudy/rainy conditions. Humidity in the air can also be detrimental to wheat pollen viability [26], but is not a concern in an indoor setting. These improvements should help wheat breeding programs that employ inter-species or inter-genus hybridization.

Evaluating wheat × rye hybrid seeds in Chinese Spring was optimal for the 1st–3rd florets in the basal and central spikelets. This result is consistent with self-pollinated seed sets. Floret fertility in the apical part of the spikelet is under the control of the *Grain Number Increase I* (*GNI1*) gene [16]. Chinese Spring harbors the reduced-function allele of *GNI-A1*, resulting in better floret fertility. It is also noted that an asymmetry and/or heterochronic floret/grain development is observed depending on the position of the florets within a spikelet; the second florets were heaviest among the florets [27,28,29].

With our new visualization technique, we successfully observed rye pollen tubes in wheat pistils. In wheat × wheat crosses, the pollen tube is not apparent due to low callose deposition; thus, we used rye pollen in this study. Rye pollen tubes from wheat × rye crosses were previously observed using the squash method [30] and paraffin sectioning [31]. The whole-mount imaging technique was recently used to observe the three-dimensional tissue organization of the Arabidopsis pistil [32] and embryo [33]. These techniques are facilitated by tissue clearing using chloral hydrate but are incompatible with observing pollen tubes using aniline blue staining. The reactivity of aniline blue fluorescence to β-glucan is optimal at a weak alkaline pH [34]. Recently developed dyes might be good candidates for improving pollen tube visualization (Table 1) [35,36]. Moreover, fluorescent proteins can be used to visualize pollen tubes, but this approach is limited to transgenic-ready plant genotypes. Two-photon microscopy has successfully been used for live imaging to trace pollen tube elongation. By contrast, our newly developed protocol is simple and can be used to conduct conventional fluorescence microscopy. Focus bracketing and stacking of images (extended depth of field) improved the resolution of pollen tube observations. Conventional single-focus imaging allowed us to focus on one site and not others. Our newly developed technique can be used to investigate various aspects of plant reproduction, including floret fertility, male sterility, and reproductive barriers in wide crosses.

In conclusion, we established effective methods for evaluating wide-crossing hybridization barriers between wheat and rye. Understanding pollen tube behavior is critical to improving hybrid seed production. Our findings provide a deeper insight into plant reproduction research and wheat breeding to enhance tolerance for global climate change.

## 4. Materials and Methods

### 4.1. Plant Materials

Bread wheat (*Triticum aestivum* L.) cultivar Chinese Spring was used as the female parent. Rye (*Secale cereale* L.) cultivar Ryetaro (Takii Seed, Kyoto, Japan) was used as the pollen parent. The plants were grown in a growth room in 11.5 cm diameter pots containing 500 mL of potting mix Tsuchitaro (Sumitomo-Ringyo-Ryokka, Aichi, Japan), 500 mL of Akadama clay (Cainz Home, Saitama, Japan), and 3 g of fertilizer (Osmocote Exact mini 16-8-11, Hyponex, Osaka, Japan). The plants were grown under a 16 h day/8 h night cycle at a daytime temperature of 22 °C supplied by LED light irradiation (360–455 μmol m^–2^ s^–1^) and a nighttime temperature of 16 °C. Floret fertility was examined using plants grown in an open field (Arid Land Research Center, Tottori University, Tottori, Japan).

### 4.2. Crossing

The first spike emergence was used to check the timing of anthesis. Spikes after the second spike emergence were used for crossing. The spikes were cut 2–3 days before anthesis, leaving two nodes. Immature spikelets from the apical/basal regions were removed, and the center parts of ten spikelets were used for crossing. The 1st and 2nd florets were used, and the remaining florets were removed. The spikes were emasculated by removing three anthers and covered with a paper bag. For spike culture, spikes were incubated in a 1.5 L PET bottle filled with 3% sucrose in a water bath cooled with 5 °C water using a chiller (EYELA CTP-3000) at room temperature (20 °C) with continuous LED irradiation. Before use, the 1.5 L PET bottle was treated with 1% bleaching agent to remove fungal contamination. A total of 20 florets were pollinated with rye pollen: half were used for pollen tube observation, and the remaining half were used to measure seed set. Pistils were collected 2 h after pollination. The spikes were cultured for 20 days in a detached spike incubator for seed development.

### 4.3. Pollen Tube Visualization

Pistils were frozen in liquid nitrogen and stored in a –80 °C freezer until use. The pistils were fixed in Carnoy’s solution (3 parts absolute ethanol: 1 part glacial acetic acid [*v*/*v*]) for 1 h. The samples were cut into sections with a knife (Feather FA-10) to observe pollen tubes at the bottom of the style. The pistils were softened in a 70% lactic acid solution in a 100 °C heat block for 2 min, soaked twice with water, transferred to 0.5 M tri-potassium phosphate, incubated for 1 h, and stained overnight in staining solution (0.1% aniline blue, 0.1 M tri-potassium phosphate, and 20 μg/mL propidium iodide [PI]). The samples were mounted on slides in mounting medium consisting of 0.4 M sorbitol and 0.1 M tri-potassium phosphate and covered with a coverslip (Matsunami C218181). The slides were observed under a BX41 fluorescence microscope (Olympus Life Science) with a U filter (excitation 330–385 nm, emission > 400 nm). Photographs were taken along the *z*-axis (16.7 μm each). An enhanced focused image was generated using Adobe Photoshop software v 26.2.0 with the automated Python “PyAutoGUI” library script. The red color channel was enhanced from 150 to 255 (a 70% increase) using “Levels” from the “Adjustments” menu. Values are shown as the average and standard deviation.

### 4.4. Graph Construction and Statistical Analysis

Graph construction and statistical analysis were conducted using R packages. The dot plot graph was created with a bandwidth of 5 using the “ggpubr” package. Significant differences among groups were determined using Tukey’s multiple comparison test with the “glut” function in the “multcomp” package and by one-way ANOVA.

## Figures and Tables

**Table 1:** Improvements related to pollen tube observation.

Objective	Method
Staining	Lactophenol-cotton blue for cellulose and callose [37] Water-soluble aniline blue for callose [38] Mixed staining with aniline blue with Calcofluor white for cellulose and callose [39] Water-soluble aniline blue and propidium iodide [this study] SR2200 for cellulose and callose [35] Pontamine fast scarlet 4B for cellulose [36] Immunostaining for pectin [36] *GUS* transgene introgression [40] Introgression of fluorescent protein transgenes [41,42]
Clearing	Sodium hydroxide [38] Lactic acid [This study] ClearSee [42]
Microscopy	Light microscopy [37] Fluorescence microscopy [38] Fluorescence microscopy with focus stacking [this study] Confocal laser-scanning microscopy [42] Two-photon excitation microscopy [41]

## Data Availability

The original data presented in the study are included in the article; further inquiries can be directed to the corresponding author.

## Supplementary Materials

The following supporting information can be downloaded at: https://www.mdpi.com/article/10.3390/plants13243600/s1, Figure S1: Fertility of self-pollinated wheat seeds at different floret positions.
