# Supplementary material for: Optimizing Visualization of Pollen Tubes in Wheat Pistils
Source: Plants (Basel). 2024 Dec 23;13(24):3600. doi: 10.3390/plants13243600 (PMC11678161; doi:10.3390/plants13243600)
Supplement: Supplementary file 1 [file plants-13-03600-s001.zip › plants-3352119-supplementary.pdf]

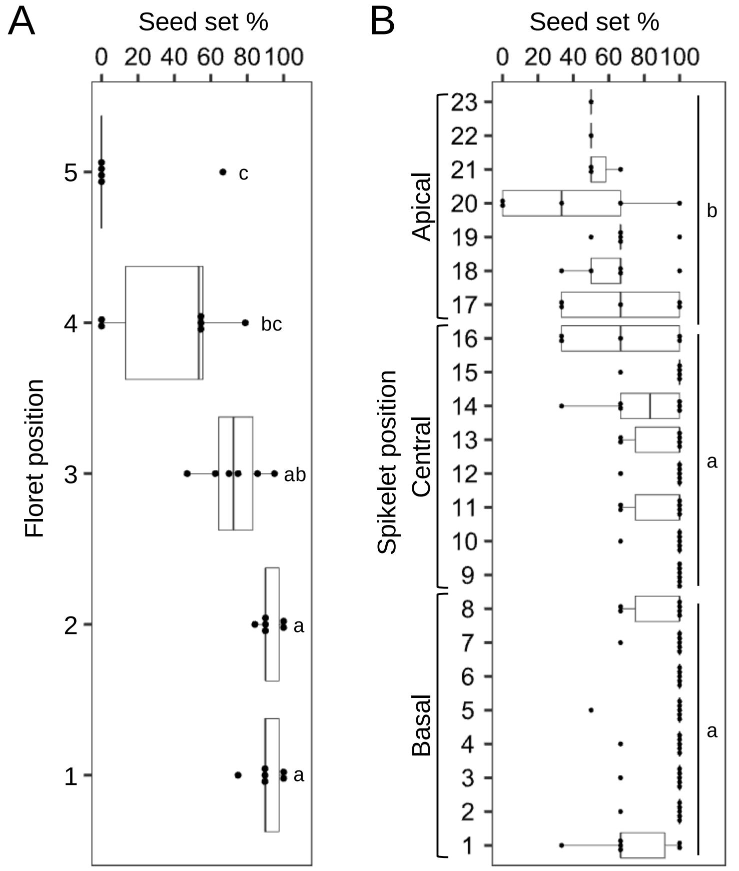

Figure S1. Fertility of self-pollinated wheat seeds at different floret positions. (**A**) Effect of floret position on seed set based on 7 spikes. (**B**) Effect of spikelet position at the 1st, 2nd, and 3rd florets on seed set based on 7 spikes. Different letters in (**A**,**B**) indicate significant differences using Tukey's multiple comparison test. Box edges represent the 25% and 75% quantiles, with the median values indicated by center horizontal lines. Whiskers indicate 1.5 times the interquartile range.
